# Sustainable Phenolic Fractions as Basis for Furfuryl Alcohol-Based Co-Polymers and Their Use as Wood Adhesives

**DOI:** 10.3390/polym8110396

**Published:** 2016-11-14

**Authors:** Paul Luckeneder, Johannes Gavino, Robert Kuchernig, Alexander Petutschnigg, Gianluca Tondi

**Affiliations:** Department Forest Product Technology & Timber Construction, Salzburg University of Applied Sciences, 5431 Kuchl, Austria; paul.luckeneder@fh-salzburg.ac.at (P.L.); johannes.gavino@fh-salzburg.ac.at (J.G.); rkuchernig.htw-m2014@fh-salzburg.ac.at (R.K.); alexander.petutschnigg@fh-salzburg.ac.at (A.P.)

**Keywords:** tannin, lignin, adhesive, furanic, poly-furfuryl alcohol, copolymer, multivariate data analysis, wood bonding, panels

## Abstract

Furfuryl alcohol is a very interesting green molecule used in the production of biopolymers. In the present paper, the copolymerization in acid environment with natural, easily-available, phenolic derivatives is investigated. The processes of polymerization of the furfuryl alcohol with: (i) spent-liquor from the pulping industry and (ii) commercial tannin from acacia mimosa were investigated though viscometry and IR-spectroscopy. The curing kinetics of the formulations highlighted the importance of the amount of furfuryl alcohol and catalyst as well as the effect of temperature for both phenolic-furanic polymers. Evidence of covalent copolymerization has been observed through infrared spectrometry (FT-IR) combined with principal component analysis (PCA) and confirmed with additional solubility tests. These bio-based formulations were applied as adhesives for solid wood and particleboards with interesting results: at 180 °C, the spent-liquor furanic formulations allow wood bonding slightly with lower performance than PVA in dry conditions, while mixed formulations allow the gluing of particleboard with only satisfactory internal bonding tests.

## 1. Introduction

Sustainable polymers are becoming more interesting because a better exploitation of green resources allows us to save crude-oil and to decrease our overall environmental impact. In this context, the furfuryl alcohol (FA) is one of the most interesting molecules in green chemistry [1,2,3]. Furfuryl alcohol is obtained by reduction of the furfural [4,5,6] which is produced from well-known bio-refinery processes starting with the hydrolysis of the hemicelluloses [7,8,9]. Its natural origin promotes this precursor for the synthesis of bio-materials even if the commercial costs of furfural (1.2–1.6 $/kg) are still high and variable because they are strongly dependent on the corn price [10,11].

One of the most intriguing reactions of furfuryl alcohol is its exothermic polymerization in acid environments [12,13]. Many studies were done for understanding this poly-condensation process which initially involves linear polymerization leading to conjugation of furanic chains and in a second stage also Diels-Alder rearrangements [14,15]. The exact structure of the poly-furfuryl alcohol is very difficult to be controlled because the polymerization occurs very rapidly in three-dimensional networking and therefore also the exact molecular conformation is hard to unscramble [16]. Poly-furfuryl alcohol (PFA) is the basis for many furanic resins which are abundantly used in blast furnaces for the production of molds for metals because of their very good thermal stability and their fire resistance at higher temperatures [13,14].

In recent years, the polymerization of furfuryl alcohol has been investigated also in combination with spent-liquor from the pulping process and tannin extracts. These two products are the two natural-based industrially-produced sources of poly-hydroxy aromatic molecules. The spent liquor is the richest fraction of lignin of the pulping process. This by-product has limited market applications and it is mostly burned to recover energy [17,18]. Conversely, the tannins are the major product of the industry which extracts them because of their broad market. Tannin extracts find application in leather tanning, adhesives, flocculants, animal feeding, enology, cosmetics, and pharmacy [19,20].

Different lignin fragments derived by various pulping processes have been combined with furfuryl alcohol to produce fire coatings, composites [21,22,23], and even adhesives [24,25].

Tannin extracts have been already investigated for polymerizing with furfural for the preparation of carbon cryogel or as an alternative adhesive component for phenolic formulations [26,27]. However, there is much more literature on tannin copolymerized with furfuryl alcohol: Copolymers with unsaturated polyesters [15] as well as natural polymers like cellulose and its derivatives [16,17] and lignin [18,19,20] were investigated with very promising results.

The latter were exploited for the synthesis of bio-based foams [28,29,30,31]. In recent years, these tannin-based foams were extensively characterized for their physical and chemical properties. They have shown limited thermal conductivities and high fire-resistance which promote them for the insulation of green buildings [32,33,34] as well as good absorbing properties against metal ions and organic pollutants [35,36]. The chemistry of this porous material was also investigated with several spectroscopic techniques to determine the covalent nature of the polymerization between the furfuryl alcohol and the tannin [37,38]. Kinetic studies with iso-conventional methods were also performed for the spruce tannin-furfuryl alcohol foams [39]. Conversely, for the lignin furanic foams and their polymers, research is still in progress and only preliminary studies were published.

Furfuryl alcohol-phenolic resins can be also proposed as an interesting alternative for replacing the synthetic, formaldehyde-based adhesives which dominate the wood industry [24,40]. In this context, these hydroxy-aromatic/furanic formulations may represent a bio-sourced, formaldehyde-free alternative to aminoplastic (Urea-Formaldehyde (UF), Melamine-Urea-Formaldehyde (MUF), Melamine-Fomaldehyde (MF)) and phenolic resins (Phenol-Formaldehyde (PF), Phenol-Resorcinol-Formaldehyde (PRF)) but also a more sustainable choice than isocyanates and polyurethanes [41,42].

The objective of the present study is the understanding of the physical and chemical properties the furfuryl alcohol blending with spent liquor from magnefite process and flavonoid tannin moieties, which are two of the most abundant, easily-available, renewable phenolic bio-resources on Earth.

Although the acid conditions required for the polymerization of the furanic polymers may degrade the wood stability [43,44], some interesting methods to neutralize the conditions post-curing were proposed [45,46] and therefore, the furanic/phenolic copolymers were also preliminarily tested as wood adhesives for wood-to-wood bonding and particleboard production to observe whether or not these polymers present promising physical properties for replacing the existing adhesives.

## 2. Materials and Methods

### 2.1. Materials

Spent liquor (SL) from the magnefite pulping process of the company Lenzing AG (Lenzing, Austria) was used. This by-product was a pH-neutralized concentrate with around 60% of solid content of which at least 60% was constituted of ligno-sulfonates. Tannin (T) powder from Mimosa (Acacia mearnsii) bark extract was supplied by Silva chimica (San Michele Mondoví, Italy). The furfuryl alcohol (FA) was supplied by Transfuran chemicals (Geel, Belgium) and the sulfuric acid 32% solution was prepared by diluting the 98% solution purchased by C. Roth (Karlsruhe, Germany).

The solid beech used for the determination of the shearing resistance of the adhesive was purchased by Holz Stefl GmbH (Kuchl, Austria) while the wood chips were the core layer ones from the industrial production of Kaindl AG (Salzburg, Austria).

### 2.2. Formulation Preparation

Spent liquor-furfuryl alcohol (SL/FA) and tannin-furfuryl alcohol (T/FA) formulations were prepared as follows: The phenolic substrates were diluted with water and added to furfuryl alcohol according to the proportions described in Table 1.

A total of 25 formulations for the spent liquor and 16 formulations for the tannin were studied. Spent liquor or tannin, water, and furfuryl alcohol were mixed applying a vigorous mechanical stirring and only when the formulation was homogeneously distributed was the sulfuric acid added.

### 2.3. Determination of Shelf- and Pot-Life

The shelf-lives of the formulations were measured producing around 50 g of SL/FA or T/FA according to the formulation in Table 1 but without adding the sulfuric acid. The viscosities of the formulations were measured the day of the preparation and after 1, 2, 3, 4, 7, 12, 16, 20, and 30 days. The pot-life were measured after adding the catalyst (according to Table 1) and the measurement were taken at regular intervals starting from the moment in which the acid catalyst was added and until complete hardening. When the formulation became solid and the viscosity was not measureable anymore, the value of 6000 was arbitrarily given. All the viscosities were measured with a rotation viscometer from Thermo Haake (Waltham, MA, USA), at room temperature (around 20 °C), inserting the spindle in a 100 mL beaker filled with the formulation under investigation.

### 2.4. Gel/Curing Time

The gel time of the SL/FA formulations were measured by dipping a glass test tube containing the all components of the formulation in Table 1 in a beaker filled with boiling water. The viscosity of the formulation was constantly monitored with a metal stick and the time until the resin turned into gel was registered as gel-time.

### 2.5. FT-IR Analysis

The resins of SL/FA 1:1 and 4:1 as well as the polymer T/FA 1:1 and 4:1 were produced with 1.5% sulfuric acid at the temperature of 100 °C. The solid spent liquor and the tannin were produced adding 1.5% sulfuric acid to a 60% solution and drying them at 100 °C for 2 h. The homo-polymer of furfuryl alcohol, the poly(furfuryl alcohol) (PFA), was produced by adding a few drops of sulfuric acid to 3 g of furfuryl alcohol. The black polymer, obtained within a few minutes through strong exothermic polymerization, was dried at 60 °C for 2 h. Every solid fraction was finely milled and scanned with a Frontier FT-IR from Perkin-Elmer (Waltham, MA, USA) equipped with ATR miracle device. The spectra were registered between 4000 and 600 cm^−1^ with 32 scans at resolution of 4 cm^−1^. Each sample was scanned three times and the average spectrum was used for this study. The software Unscrambler X (Camo) (Oslo, Norway) was used for the analysis of data. The spectra were all studied in the region between 1800 and 600 cm^−1^, baseline corrected, and area normalized before being elaborated for the principal component analysis (PCA) performed with the algorithm NIPALS (NonLinear Iterative Partial Least Squares).

### 2.6. Water Solubility Test/Leaching Resistance

The seven solids prepared for the FT-IR analysis were leached with the following method: around 1.0 g of powder was mixed with 200 mL of deionized water in a 250 mL beaker and kept under magnetic stirring for 1 h at room temperature. The suspensions obtained were filtered and the filter was dried overnight at 103 °C. The final weight was registered and the insoluble fraction (If) was calculated according to the following formula.

$$If\left(\%\right)=\frac{W_{a}}{W_{b}}\times 100$$

where *W*_a_ and *W*_b_ are the weight after and before leaching, respectively.

The percentage of estimated insoluble (*EI*) was calculated with the following formula:

$$EI\;(\%)=(\frac{X_{\mathrm{fa}}}{100}\times If_{\mathrm{pfa}})+\left(\frac{X_{\mathrm{phen}}}{100}\times If_{\mathrm{phen}}\right)$$

where *X*_fa_ and *X*_phen_ are the percentage of furfuryl alcohol and phenolic in the formulation while *If*_pfa_ and *If*_phen_ are the material left of the PFA and phenolic (SL or T), respectively.

The percentage of phenolic polymerized (*PP*) was calculated as follows:

$$PP\;\left(\%\right)=\frac{If-EI}{X_{\mathrm{phen}}}\times 100$$

### 2.7. Solid Wood Gluing and Shearing Tests

Two beech lamellas of 700 × 150 × 5 mm^3^ were glued with 250 g/m^2^ of SL/FA or T/FA adhesive and cured at different temperatures from 120, 150, and 180 °C. Commercial poly-vinylacetate adhesive at room temperature and solid beech samples were used as control. The glued lamellas were cut into 70 × 15 × 10 mm^3^ pieces and mechanically tested according to DIN EN 302-1 [47]. For the most promising formulations, the test was repeated after 24 h soaking in cold water. The specimens were tested with a Zwick Roell 250 (Ulm, Germany) universal testing machine with 2 mm/min. The maximal strength and the % of broken wood in the bondline area were measured.

### 2.8. Particleboard Production

SL/FA and T/FA adhesives were used to glue industrial core layer particles with the procedure described elsewhere [48]. The particles and the adhesive (10% or 15% by weight) were mixed in a ploughshare mixer for 30 s at 200 rpm. The glued chips were distributed over a wooden mold of 500 × 500 mm^2^ and pressed with a HLOP 280 press from Hofer (Innkreis, Austria) to a final height of 19 mm and with target density of 650 kg/m^3^ at platen temperature of 180 °C for 6 min. The so-made panels were stabilized at 20 °C and 65% moisture content (m.c.) for one week before being cut for testing.

### 2.9. Density and Density Profile

The normal density (*d*) of every panel was measured by registering the mass (*m*) and the volume (*V*) of each panel and applying the formula $d=\frac{m}{V}$; while the density profile was measured for three specimens of 50 × 50 × 19 mm^3^ using a Dense-lab X from EWS (Hameln, Germany). The data are reported as average and corrected by % along the thickness.

### 2.10. Mechanical Properties

Internal bond and three point bending tests were performed for each panel produced. Five specimens with a size of 50 × 50 × 19 mm^3^ were tested for their internal cohesion according to DIN EN 310 [49] while three specimens of 400 × 50 × 19 mm^3^ were tested according to DIN EN 312 [50] for their modulus of elasticity (MOE) with a universal testing machine Zwick Roell Z 250 (Ulm, Germany) with a testing rate of 2 mm/min.

## 3. Results

### 3.1. Curing Process Characterization

The present paper describes the way of preparing furfuryl alcohol-based resins in which the aromatic counterparts are: (i) the lignin moieties directly obtained from the magnefite pulping (SL/FA) and (ii) the industrial extract of acacia mimosa tannin (T/FA). The first step of our investigation was the viscoelastic monitoring of the properties of the different furanic/phenolic formulations.

**Figure 1:**
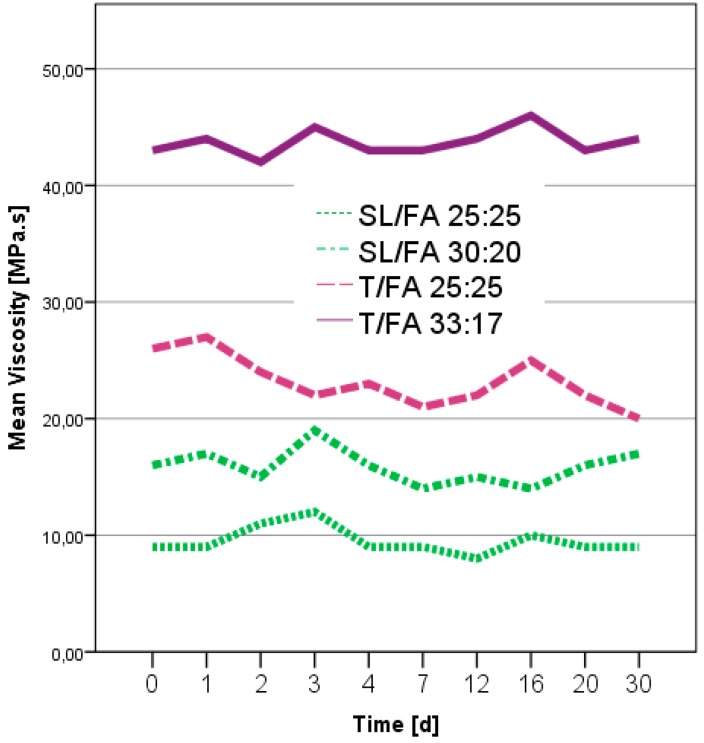
Viscosity trend of spent-liquor/furfuryl alcohol and tannin/furfuryl alcohol formulations.

Initially the shelf life, which is the time that a formulation can be stored without changing its properties, was determined. In Figure 1 the viscosities of the different formulations during one month from the date of the preparation are reported.

The viscosities of every furfuryl alcohol-based formulations with different ratios of spent liquor/FA and tannin/FA showed no significant modifications within the first 30 days of storage. Particularly interesting was that tannin formulations, which usually produce a skin on their top after a few hours, remained as a viscous liquid once the furfuryl alcohol was added.

**Figure 2:**
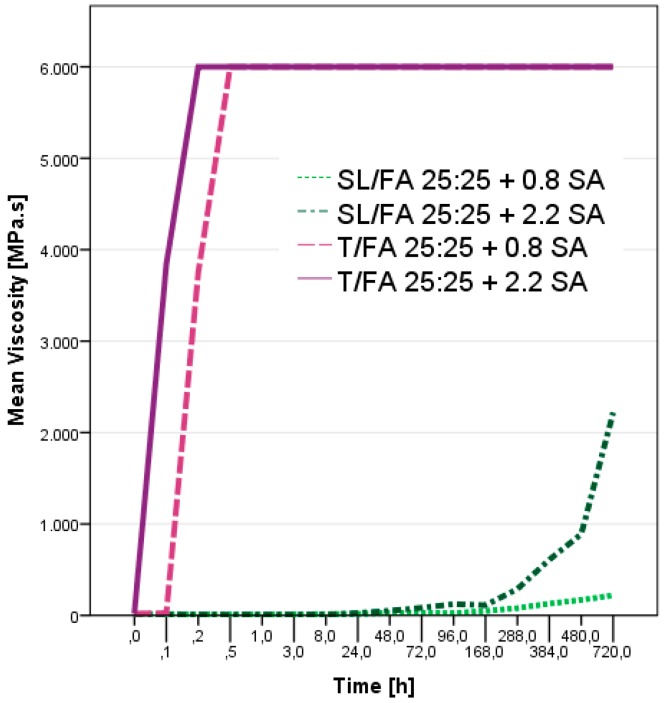
Viscosity behavior of spent-liquor/FA and tannin/FA formulations after addition of different amount of sulfuric acid (SA) as hardener.

The behavior of the phenolic-furanic formulations changed when already 0.8% of acid was added. The time after addition of the acid catalyst, namely the pot-life, was observed for the formulation having higher amount of FA (SL/FA 25:25 and T/FA 25:25) and the results are presented in Figure 2.

**Figure 3:**
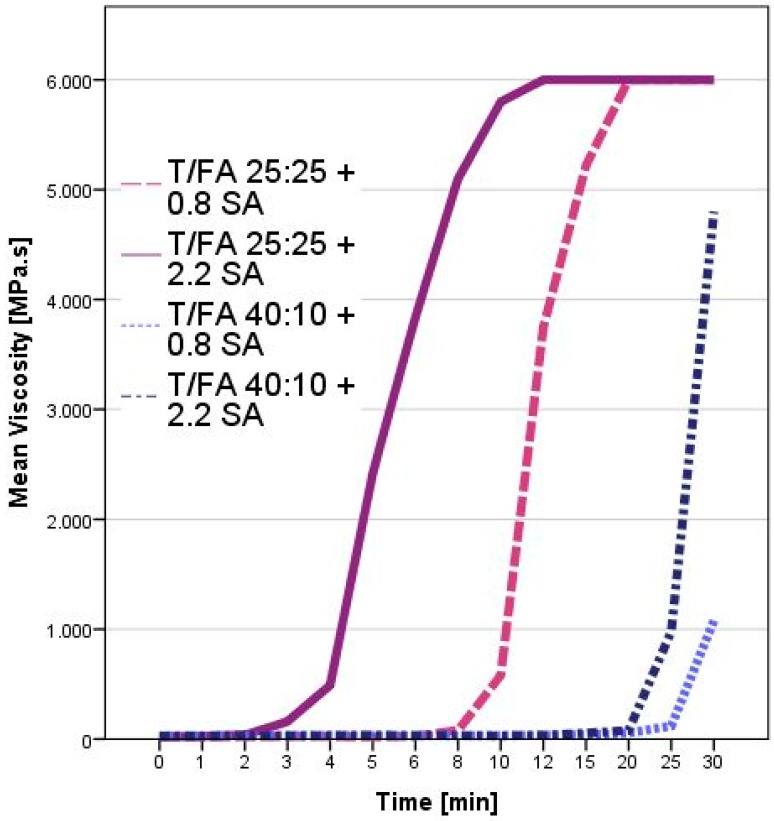
Viscosity behavior of tannin-FA formulations depending on the ratio T:FA and on the amount of sulfuric acid (SA) as catalyst.

The behavior of the two hydroxy-aromatic substrates was different. The spent-liquor/FA formulations increased their viscosity very slightly and they needed several days before observing a significant viscosity increase. After 30 days, even the formulation catalyzed by high amount of sulfuric acid (2.2%) did not reach the solid state yet. Conversely, the corresponding tannin/FA formulations were solid after 20 min of reaction. For this substrate, the effect of the amount of sulfuric acid was more evident and the curing occurred rapidly. In order to better understand the hardening process of tannin/FA at room temperature, the viscosity was observed more into details also for the formulations with lower proportion of furfuryl alcohol. In Figure 3, the viscosity trend for four T/FA formulations are reported.

The kinetic of curing of the tannin-FA formulation can be well understood from this graphic because the importance of having FA and sulfuric acid are elucidated. Both components are fundamental for the polymerization process: (i) 0.8% of sulfuric acid is necessary to start the reaction and (ii) FA is the active component which greatly affects polymerization. However, once the conditions for starting the reaction are satisfied, the amount of FA has a greater impact for the curing of the resin than the amount of catalyst. It has to be considered that the polymerization of furfuryl-alcohol in presence of phenolic counterparts is temperature-dependent: higher temperature contributes to speed up the curing. So, once the reaction was started, the exothermic polymerization reaction increased the temperature of the bulk and led to the exponential trends observed for every formulation.

In order to get more information for the SL/FA formulations, the behavior at higher temperature were observed through the gel-time test. In Table 2, the gel time in seconds of the SL/FA formulations are presented.

The table shows that also for the formulations with spent liquor as aromatic building block, the amount of furfuryl alcohol and the amount of catalyst used strongly affects the reaction rate. However, the formulation SL/FA 30:20 is the faster curing than the SL/FA 25:25. This phenomenon is due to the different initial viscosities of the lignin-furanic formulations. The ones with more furfuryl alcohol were more liquid at the beginning of the process and so the gel time occurred slightly later. It is worth reporting that, after 30 min at 100 °C, only the formulations having at least 20% of furfuryl alcohol (% *w/w* total formulation) were completely solid, all the others increased further their viscosity but remained in a viscous liquid state.

The furfuryl alcohol-based resins have shown the potential to cure with both the phenolic building-blocks. With tannin stable solids were obtained already at room temperature, while for the spent-liquor the external support of heat was necessary to allow the hardening to occur quickly.

**Figure 4:**
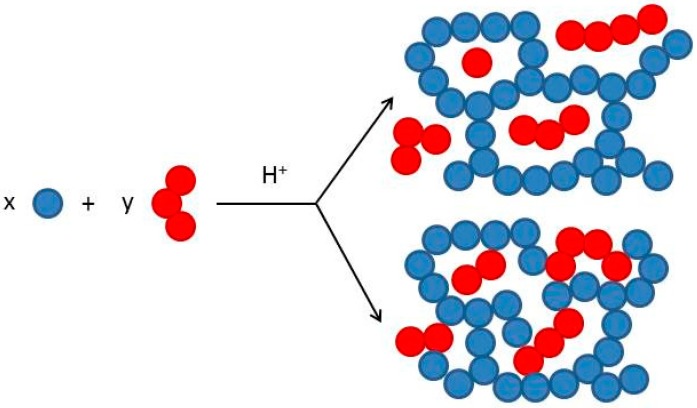
Possible reaction mechanisms between furanic unit (**Blue**) and phenolic moieties (**Red**).

The major question to be considered about these adducts is whether or not they are covalently combining. Two phenomena may occur: (i) The molecules of the phenolics (red) remain trapped into the furanic structure (blue); (ii) the phenolics and furanic units are covalently combined (Figure 4).

**Figure 5:**
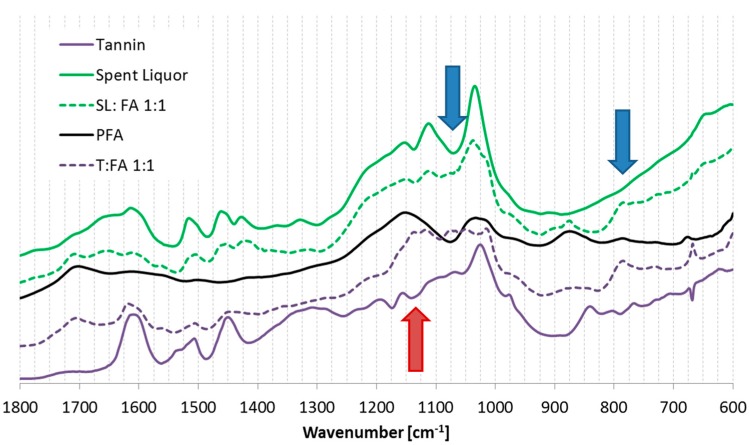
FT-IR spectra of spent liquor- and tannin-furanic solids: Spent liquor (**Green bold**); SL:FA 1:1 (**Green dotted**); Poly furfuryl alcohol (**Black bold**); Tannin (**Purple bold**); and T:FA 1:1 (**Purple dotted**).

The hardened solids were investigated with the FT-IR to understand if any evidence of covalent polymerization can be observed between the furanic and the phenolic part. The spectra of spent liquor (SL/FA) and tannin (T/FA) furanic powders are reported in Figure 5.

In this figure, it can be observed that almost all of the signals of the mixtures are intermediate between the signals of the phenolic resource (SL and T) and the ones of PFA.

However, the mixture presents a couple spectral regions in which the signals observed are different to the ones expected: in case of spent liquor-furanic powders the two most important differences occurs at 1070 and 790 cm^−1^ (blue arrows) (the band at 1420 cm^−1^ is just slightly shifted); while for tannin-furanic powders, the whole region between 1140 and 1050 cm^−1^ (red arrow) and the signal at 790 cm^−1^ present a clear distance from the expected.

According to literature, the region between 1140 and 1000 cm^−1^ is due to C–H and C–O vibrations and the region around 790 cm^−1^ is due to aromatic ring torsions [51,52]. Hence, new vibrations related to C–H and C–O bonding are observed for both phenolics: in case of SL/FA only contained modification is observed; while in case of T/FA more significant evidences are visible.

**Figure 6:**
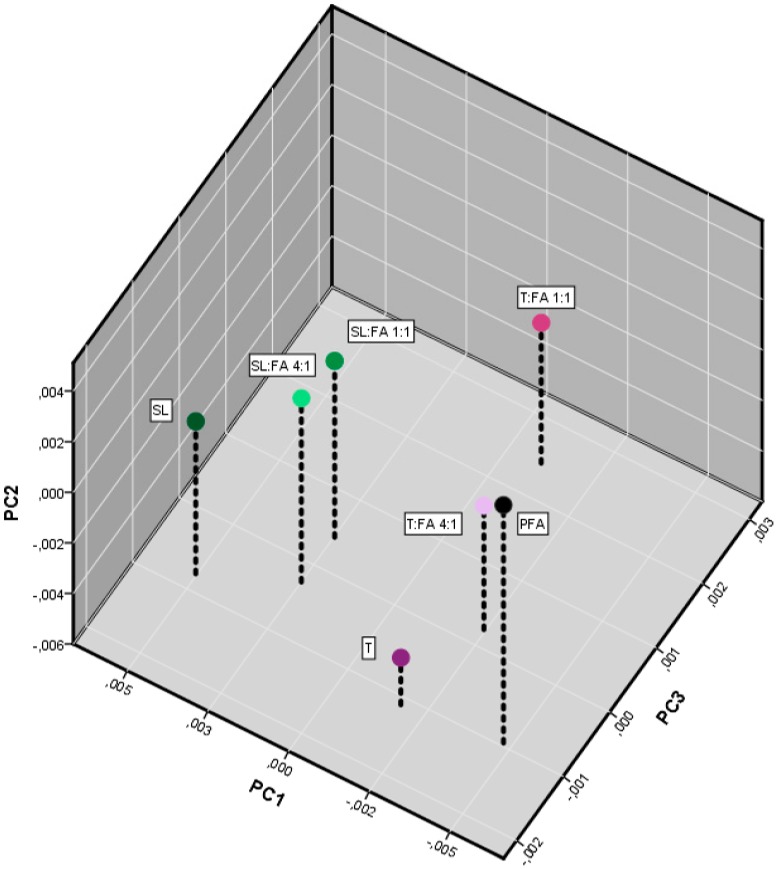
Principal component analysis of the FT-IR spectra of the furanic-phenolic polymers.

In order to extrapolate further information, the analysis of the principal components was also performed and the three main principal components were plotted in a 3D graphic reported in Figure 6.

This graphic shows that the three precursors (PFA, SL, and T) stay in the vertex of a triangle in the plan defined by the two principal components (PC1 = 59% and PC2 = 27%). In case the components do not interact, it would be expected that the cured mixture as well would lay in this plane; conversely the lignin- and the tannin-furanic blends stay out of the plane proportionally to the amount of furanic units. The more furanic moieties are included in the copolymer, the more the PC3 (8%) will be affected. Furthermore, the tannin (T/FA) resins, which are more reactive, are found at higher distances than the homologues spent liquor (SL/FA). This may suggest that the PC3 carries the most interesting information about the formation of new bonds.

**Figure 7:**
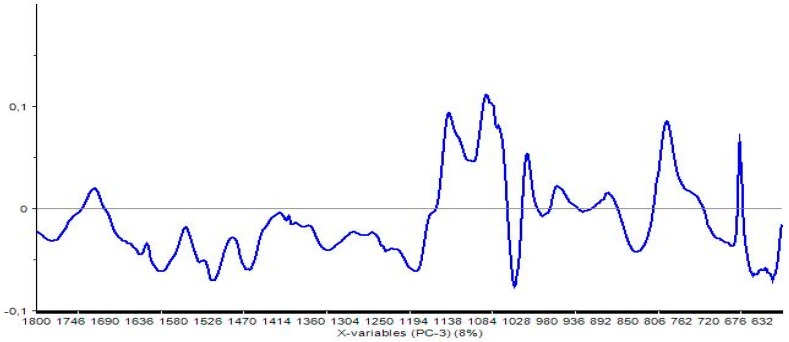
Loadings of the principal component 3 (PC3).

If we look at the loadings of PC3 (see Figure 7) it can be observed why the signals which cannot be explained as overlapping of the polymers, are more interesting for the polymerization process.

The loadings in the region between 1140 and 1050 cm^−1^ and that at around 780 cm^−1^ are the most important. These loadings explain that the PC3 is the component that results more sensitive to the presence of new signals even if its weight is only 8%.

The polymerization, indeed, seems to be covalent for both substrates. However, for T/FA the bonding between tannins and furanic units are numerically higher than the connections between the lignin moieties of the spent liquor and the furanic chains.

This evidence of molecular combination will further confirm the study of Nordstierna et al. which proposed interconnection between lignin and poly(furfuryl alcohol) [53].

A further test which allowed us to obtain further insights about the phenolic/furanic co-polymerization was the water solubility test. The powders prepared for FT-IR investigation were leached and their solubility performances are reported in Table 3.

The table shows that acidified spent liquor and tannin remain almost completely soluble, while the poly-furfuryl alcohol is very insoluble (93.8% insoluble and transparent leaching water). The phenolic/furanic powders present intermediate leaching resistances.

The estimated insoluble amount is calculated assuming that no interaction occurs in the blends—e.g., the SL/FA 4:1 blend is constituted by 20% of furanics (93.8% insoluble) and 80% of spent liquor (2.2% insoluble) and hence the estimated insoluble is 20.5%. In reality, the insoluble amount is much higher. Therefore, we can state that a certain portion of the original phenolics combines with the furanic matrix and its quantification is shown in the fifth column of Table 3. According to this idea, it looks that around one-third of the original lignin powder reacts with the furanic for formulation 4:1, but up to more than half can be fixed when furfuryl alcohol in proportion 1:1 is considered. In case of tannin, the polymerization is more complete independently on the amount of furfuryl alcohol applied and goes from 85.9 (for T/FA 4:1) up to 88.8 (for T/FA 1:1). Although the use of other investigation techniques such as GPC, XRD, and solid state ^13^C-NMR will allow determination of further copolymerization details, the present investigation suites to identify the interaction between furanic and phenolic moieties.

There are three reasons to be considered for explaining why the condensed tannins are more reactive than spent liquor in the copolymerization process with furanic resins: (i) The condensed tannins are more reactive than lignin in polymerization processes [54]; (ii) The tannin extract is constituted of at least 80% of poly-phenols while the spent liquor contains around 60% of lignosulphonates; (iii) The non-phenolic part for tannin is constituted principally of hydrocolloids which can be more easily combined in the furanic network than the non-phenolic of the spent liquor which are constituted of low-molecular sugars and even inorganic salts. Summarizing, the co-polymerization of tannin with furfuryl alcohol was relatively easy to observe: the FT-IR present new signals and the powder solubilizes around 10% which might be due to hydrocolloids or unreacted low molecular mass fractions. Conversely, the spent liquor/furanic co-polymers were more difficult to detect by FT-IR but the information collected was congruent with solubility tests which suggest the copolymerization of these species albeit to a more contained extent.

### 3.2. Adhesive Properties

The furanic-phenolic formulations have shown defined curing properties and therefore they were investigated for their capacity to glue solid wood and particleboards.

#### 3.2.1. Solid Wood Gluing: Shearing Tests

The furfuryl alcohol-based resins were tested for gluing solid wood and their performances are summarized in Table 4. According to the kinetic study presented, it has to be considered that the furanic-based formulations for wood gluing are difficult to prepare because: (i) the presence of different amount of components as well as (ii) the preparation and application time, play major roles on the viscosity of the adhesive which could prejudice the efficacy of the bondline.

The SL/FA formulations presented a broad range of results. The formulation containing limited amount of furfuryl alcohol (40:10), limited amount of catalyst (<2%) and glued at lower temperature (<120 °C) allowed no/very weak bonding. Conversely, the formulations richer in furfuryl alcohol (25:25) and glued at higher temperature (180 °C) presented good bonding which reached almost the same performances as the commercial polyvinylacetate with solid wood breaks higher than 90%. According to these data, ideal formulations need to be cured at 180 °C and must have a maximal ratio SL:FA of 30:20. These formulations did not resist immersion in water.

Also for the T/FA resins, the higher gluing performances occurred when at least 2.2% of catalyst and at 180 °C were applied. However, these formulations allowed good results also with lower proportions of furfuryl alcohol. The most interesting finding was that the tannin-furanic formulations offered even a certain water resistance.

#### 3.2.2. Particleboard Production

**Figure 8:**
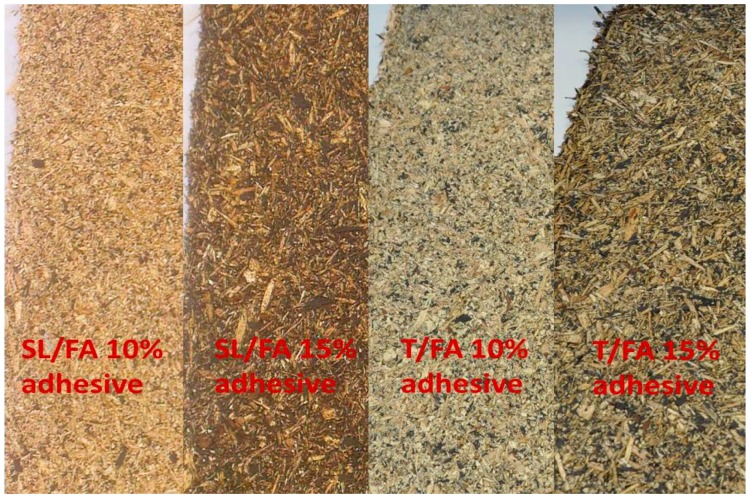
Particleboards: Spent-Liquor/Furfuryl alcohol with 10% and 15% of glue (**left side**) and Tannin-Furfuryl alcohol with 10% and 15% glue (**right side**).

**Figure 9:**
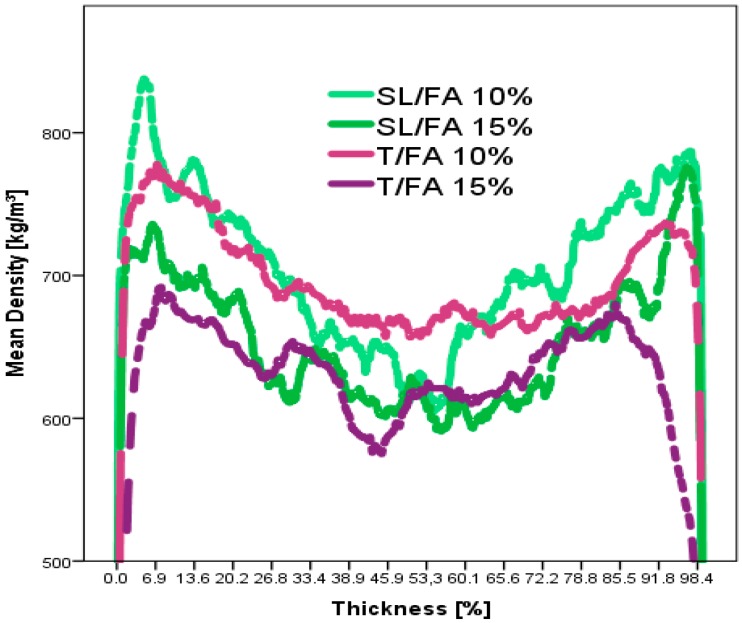
Density profile of the particleboards with spent-liquor/FA (10% and 15%) and tannin/FA (10% and 15%).

Several panels were produced with different formulations and amounts of glue. The appearance and the density profiles of SL/FA and T/FA boards are reported in Figure 8 and Figure 9.

It can be observed that the T/FA panels present regions in which black adhesive spots are accumulating and clearer wood chips compared to the SL/FA ones. Indeed, the lower reactivity of the SL/FA formulation allows a better distribution of the glue over the panel (Figure 8). However, this apparent inhomogeneity could not be observed along the thickness of the boards which presented the typical profile of the ideal particleboards where the top and bottom part have higher local densities, while the core result lighter (Figure 9).

The prepared panels were mechanically tested for their mechanical properties, the internal bond, and the bending strength. The results are resumed in the following Table 5.

The synthesis of furanic-phenolic adhesive for particleboard is also very sensitive. Higher amounts of catalyst are required so that the resin can be hardened homogeneously and as soon as the acid is added the polymerization of the resin starts even before the press stage (significantly for the T/FA formulations).

As logically expected, the particleboards produced with 10% of adhesive show weaker mechanical properties than the ones produced with 15%. It was observed that the production of panels with pure SL/FA and pure T/FA present weaker mechanical properties than the mixed formulations. In particular, it can be observed that mixed SL:T 50:10 and 40:20 reach internal bonds higher than 0.5 MPa and elastic modulus higher than 10 MPa. We can consider that the spent liquor allows a better adhesive distribution and slows down the curing of the phenolic-furanic adhesive. These performances are encouraging considering that the requirements for internal bond for dry usage (P4) are satisfied (target: 0.35 MPa), even if the bending strength is not acceptable yet (target: 15 MPa) [50]. However, these performances are superior to the one observed for particleboard produced with similar formulations in alkaline pH [55]. The cured furanic co-polymers are, indeed, very rigid and then enhancement of their performance as adhesive will be obtained as soon as their elasticity increases.

While the SL/FA glued particleboards did not resist water at all, the T/FA ones glued with 10% and 15% of resin showed a thickness swelling of 135% and 85%, respectively. This means that both formulations are very water sensitive and hence these adhesives are not be suitable for any application in contact with water.

## 4. Conclusions

Furfuryl alcohol copolymers with easily-available phenolic counterparts have been produced under different proportions, acidity, and temperature conditions. It was observed that the tannin/furfuryl alcohol polymers (T/FA) are much faster curing than the spent liquor/FA copolymers. Both phenolic substrates provided a shelf life longer than 30 days. The IR spectroscopy and the principal component analysis combined with the solubility tests highlighted that the furanic moieties copolymerize with both phenolic fractions: in particular, the tannin polymerize to a higher extent than the spent liquor. More detailed information about the copolymerization process could be gained via GPC, XRD, and solid-state ^13^C-NMR techniques.

The SL/FA and the T/FA copolymers have been tested for their gluing properties and it was observed that satisfactory solid wood bonding occurs when high temperatures are used for curing. The use of mixed resin between T/FA and SL/FA were showing the higher performances for particleboard production because they ensured good internal cohesion even if limited bending properties were still registered. Summarizing, the furfuryl alcohol copolymers are very hard to handle because of their complicated kinetics. Their polymerizations are hard to control because relatively small variations on the amount of furfuryl alcohol, acid catalyst, and slightly different temperature and time affect the viscoelastic properties of the polymer. However, once these technological drawbacks will be solved through automatized industrial process and/or by putting in specific additives for: increasing elasticity, alkalinizing the pH and improving water resistance; then these formulations will deserve to be reconsidered for their application in wood adhesive technology.

## Figures and Tables

**Table 1:** Composition of spent-liquor/furfuryl alcohol (SL/FA) and tannin/furfuryl alcohol (T/FA) formulations.

**Spent liquor (SL) %**	**Furfuryl alcohol (FA) %**	**Sulfuric acid (SA) %**	**Water %**
45	5	0–0.8–1.5–2.2–2.8	47.2–47.8–48.5–49.2–50
40	10	0–0.8–1.5–2.2–2.8	47.2–47.8–48.5–49.2–50
35	15	0–0.8–1.5–2.2–2.8	47.2–47.8–48.5–49.2–50
30	20	0–0.8–1.5–2.2–2.8	47.2–47.8–48.5–49.2–50
25	25	0–0.8–1.5–2.2–2.8	47.2–47.8–48.5–49.2–50
**Tannin (T) %**	**Furfuryl alcohol (FA) %**	**Sulfuric acid (SA) %**	**Water %**
45	5	0–0.8–1.5–2.2	47.8–48.5–49.2–50
40	10	0–0.8–1.5–2.2	47.8–48.5–49.2–50
33	17	0–0.8–1.5–2.2	47.8–48.5–49.2–50
25	25	0–0.8–1.5–2.2	47.8–48.5–49.2–50

**Table 2:** Gel-time in seconds of SL/FA formulations hardened with different amount of catalyst at 100 °C.

Formulation	Amount of H_2_SO_4_		
	0.8%	1.5%	2.2%
SL/FA 45:5	860	380	300
SL/FA 40:10	520	460	290
SL/FA 35:15	600	360	316
SL/FA 30:20	122	60	46
SL/FA 25:25	250	78	74

**Table 3:** Insoluble fraction, estimated insoluble, phenolic insoluble, and phenolic polymerized expressed in %.

Powder	Color of the leaching water	Insoluble fraction (%)	Estimated insoluble (%)	% of phenolic polymerized
Spent Liquor	Intense Brown	2.2 (0.21)	(2.2)	-
Tannin	Intense Brown	1.6 (0.35)	(1.6)	-
PFA	Transparent	93.8 (3.93)	(93.8)	-
SL/FA 4:1	Intense Brown	47.9 (3.17)	20.5	34.2
SL/FA 1:1	Brown	76.1 (3.80)	48.0	56.2
T/FA 4:1	Pale Brown	88.7 (2.21)	20.0	85.9
T/FA 1:1	Pale Brown	92.1 (2.27)	47.7	88.8

**Table 4:** Solid wood gluing: Shearing test results listed according to formulation and curing condition.

Formulation	Temperature (°C)	Shearing strength (N/mm^2^)	Standard deviation	% of Broken wood
SL/FA 35:15 + 2.2 SA	120	0	0	0
SL/FA 35:15 + 2.2 SA	150	9.4	1.37	11
SL/FA 35:15 + 2.2 SA	180	12.1	1.37	75
SL/FA 30:20 + 2.2 SA	120	8.2	3.31	0
SL/FA 30:20 + 2.2 SA	150	9.9	1.36	48
SL/FA 30:20 + 2.2 SA	180	14.1	1.33	97
SL/FA 25:25 + 2.2 SA	120	11.4	2.51	78
SL/FA 25:25 + 2.2 SA	150	12.6	1.59	63
SL/FA 25:25 + 2.2 SA	180	15.0	1.02	96
SL/FA 25:25 + 2.2 SA *	180	0	0	0
T/FA 40:10 + 2.2 SA	180	11.5	0.49	100
T/FA 40:10 + 2.2 SA *	180	1.9	0.50	60
T/FA 25:25 + 0.8 SA	180	0	0	0
T/FA 25:25 + 2.2 SA	120	0	0	0
T/FA 25:25 + 2.2 SA	180	12.7	1.29	100
T/FA 25:25 + 2.2 SA *	180	2.9	0.85	60
Polyvinylacetate	-	16.6	1.41	100
Solid wood	-	16.6	2.68	100

* Results after water storage for 24 h.

**Table 5:** Mechanical performances of the SL/FA and T/FA particleboard test.

Formulation of the glue (Dry)							
Spent liquor (%)	Tannin (%)	FOH (%)	H_2_SO_4_ (g)	Amount of adhesive %	Density (kg/m³)	Internal bond (MPa)	Bending E-Mod (MPa)
60	-	40	3.8	10	744.3	0.26 (0.06)	6.46 (0.40)
50	10	40	3.8	10	750.8	0.32 (0.03)	7.56 (0.52)
40	20	40	3.8	10	735.3	0.32 (0.06)	6.47 (1.86)
-	60	40	3.8	10	715.8	0.15 (0.05)	9.71 (2.10)
60	-	40	3.8	15	677.8	0.35 (0.14)	11.35 (1.35)
50	10	40	3.8	15	691.6	0.55 (0.16)	10.81 (1.24)
40	20	40	3.8	15	659.4	0.53 (0.16)	9.49 (1.86)
-	60	40	3.8	15	644.7	0.07 (0.03)	6.41 (0.25)
